# The interaction between 5-HTTLPR and stress exposure influences connectivity of the executive control and default mode brain networks

**DOI:** 10.1007/s11682-016-9633-3

**Published:** 2016-10-13

**Authors:** Dennis van der Meer, Catharina A. Hartman, Raimon H. R. Pruim, Maarten Mennes, Dirk Heslenfeld, Jaap Oosterlaan, Stephen V. Faraone, Barbara Franke, Jan K. Buitelaar, Pieter J. Hoekstra

**Affiliations:** 1 Department of Psychiatry, University Medical Center Groningen, University of Groningen, P.O. Box 30001, 9700 RB, Groningen, The Netherlands; 2 Department of Cognitive Neuroscience, Donders Institute for Brain, Cognition and Behaviour, Radboudumc, Nijmegen, The Netherlands; 3 Centre for Cognitive Neuroimaging, Donders Institute for Brain, Cognition and Behaviour, Radboudumc, Nijmegen, The Netherlands; 4 Department of Clinical Neuropsychology, VU University Amsterdam, Amsterdam, the Netherlands; 5 Departments of Psychiatry and of Neuroscience and Physiology, SUNY Upstate Medical University, Syracuse, USA; 6 K.G. Jebsen Centre for Psychiatric Disorders, Department of Biomedicine, University of Bergen, Bergen, Norway; 7 Departments of Human Genetics and Psychiatry, Donders Institute for Brain, Cognition and Behaviour, Radboudumc, Nijmegen, The Netherlands; 8 Karakter Child and Adolescent Psychiatry University Centre, Nijmegen, The Netherlands

**Keywords:** Serotonin transporter, Psychological stress, Attention-deficit/hyperactivity disorder, Gene-environment interaction, Functional magnetic resonance imaging

## Abstract

We recently reported that the *serotonin transporter* polymorphism *5-HTTLPR* moderates the relation between stress exposure and attention-deficit/hyperactivity disorder (ADHD) severity. This gene-environment interaction (GxE) has been previously tied to the processing of emotional stimuli, which is increasingly recognized to be a key factor in ADHD-related impairment. The executive control and default mode brain networks play an important role in the regulation of emotion processing, and altered connectivity of these networks has also been associated with ADHD. We therefore investigated whether resting-state connectivity of either of these networks mediates the relation of *5-HTTLPR* and stress exposure with ADHD severity. Resting-state functional magnetic resonance imaging, genetic, and stress exposure questionnaire data was available for 425 adolescents and young adults (average age 17.2 years). We found that *5-HTTLPR* S-allele carriers showed a more negative relation between stress exposure and connectivity of the executive control network than L-allele homozygotes, specifically in the pre/postcentral gyrus, striatum, and frontal pole. In the default mode network, we found a positive association between the GxE and supramarginal gyrus connectivity. Connectivity of either network did not significantly mediate the effect of this GxE on ADHD. Opposite effects of stress exposure on connectivity in the executive and default mode networks may contribute to findings that stress exposure is associated with lowered cognitive control and heightened levels of rumination and worrying, for S-allele carriers but not L-allele homozygotes. When combined, these effects on connectivity of both networks may relate to the emotional problems seen in individuals with ADHD.

**Electronic supplementary material:**

The online version of this article (doi:10.1007/s11682-016-9633-3) contains supplementary material, which is available to authorized users.

## Introduction

Our genetic make-up influences how we respond to environmental factors. The study of genes moderating the effect of environmental risk factors for psychiatric disorders may therefore lead to a better understanding of the etiology of these disorders than studying genetic and environmental factors in isolation (Caspi and Moffitt 2006). The most investigated gene-environment interaction (GxE) in psychiatry is the interaction between a polymorphism in the promoter region of the serotonin transporter gene (*5-HTTLPR*) and exposure to psychosocial stress. Numerous papers have reported that people carrying the short variant (S-allele) of *5-HTTLPR* have a stronger association between stress exposure and psychiatric disorders than people homozygous for the long variant of this gene (L-allele; Caspi et al. 2010), and animal models have provided evidence of a causal relation of this GxE with a range of pathological behaviors (Spinelli et al. 2007).

We recently found that S-allele carriers have a stronger relation between stress and ADHD severity than L-allele homozygotes, independent of internalizing comorbidity (van der Meer et al. 2014). On the other hand, a meta-analysis has found that on average L-allele homozygotes have a higher risk of ADHD (Gizer et al. 2009). S-allele carriers and L-allele homozygotes differ in the detection and subsequent processing of stimuli, particularly emotional ones, which may explain the role of *5-HTTLPR*, and its interaction with stress exposure, in ADHD. Several studies have shown that S-allele carriers outperform L-allele homozygotes on a range of neuropsychological tasks (Borg et al. 2009; Roiser et al. 2007), while others have rather reported lower performance (Owens et al. 2012; Fischer et al. 2015). Higher sensitivity to stimuli by S-allele carriers may explain the heterogeneity of findings, as this would be beneficial under positive conditions while having a negative effect under stressful, emotional conditions (Homberg and Lesch 2011). S-allele carriers show higher levels of neuroticism (Lesch et al. 1996), rumination (Clasen et al. 2011), and cognitive reactivity (Wells et al. 2010), a tendency for dysfunctional thinking when exposed to stressors (Barnhofer and Chittka 2010). Combined with an often-reported attention bias to negatively-valenced information (Pergamin-Hight et al. 2012), these differences in the processing of stimuli are likely to contribute to the stronger relation between stress exposure and psychopathology for S-allele carriers than for L-allele homozygotes. There is considerable overlap between the behavioral correlates of the S-allele and ADHD; results from a range of studies suggest that individuals with ADHD are also more sensitive to stimuli. They have been shown to respond stronger to positive reinforcement than healthy individuals (Luman et al. 2005), but also to have a tendency to orient less towards positive than negative emotional stimuli (Shaw et al. 2014), to be distracted more by negative emotional information in cognitive tasks (Posner et al. 2011), to score higher on measures of neuroticism (Martel et al. 2009; Parker et al. 2004), and to have more negative automatic thoughts (Mitchell et al. 2013).

Neuroimaging studies of *5-HTTLPR,* and its interaction with stress, have provided clues which neural pathways may mediate the effects of this GxE on behavior. The majority of studies into *5-HTTLPR* have employed a region-of-interest approach, focusing on the activity of the amygdala and associated limbic regions when exposed to an acute stressor. These studies have mostly found higher activity of these regions in S-allele carriers (Munafo et al. 2008). Pezawas et al. reported that the association between *5-HTTLPR* and anxiety is partly explained by lowered top-down control of the anterior cingulate cortex over the amygdala when faced with stressful stimuli (Pezawas et al. 2005). Whole-brain investigations have reported that S-allele carriers show stronger effects of long-term stress exposure on the structure and activity of frontal brain regions, particularly the anterior cingulate cortex, than L-allele homozygotes (Canli et al. 2006; Selvaraj et al. 2011). In line with this literature, we recently reported that gray matter volume in the anterior cingulate cortex and frontal pole mediates the association of the interaction between *5-HTTLPR* and stress exposure with ADHD severity (van der Meer et al. 2015). Together, these findings suggest that the heightened sensitivity to stress by S-allele carriers may involve both enhanced emotional reactivity by limbic structures and lower cognitive control by frontal cortical structures.

Our understanding of the neural mechanisms underlying *5-HTTLPR*, and its interaction with stress exposure, may be increased by studying measures of functional network connectivity, as proper serotonin signaling has been shown to be crucial for the development of fundamental neural networks (Sodhi and Sanders-Bush 2004). Functional connectivity networks are sets of brain regions that have highly correlated activity patterns, thought to reflect a shared function. Through independent component analysis (ICA), Smith et al. have identified a set of intrinsic connectivity networks during resting conditions which could be unambiguously matched to brain networks found when averaging over activity patterns from a large amount of functional magnetic resonance imaging (fMRI) studies during a range of tasks (Smith et al. 2009), thereby providing a wealth of information on their behavioral correlates. These networks can be identified with high reliability and replicability (Damoiseaux et al. 2006). Further, network connectivity measures have been postulated to better account for behavioral effects of genetic variation than local measures of brain activity or structure (Meyer-Lindenberg 2009), and have proven to be a powerful tool in the study of psychiatric disorders, including ADHD (Oldehinkel et al. 2013; Broyd et al. 2009).

Given that differences between S-allele carriers and L-allele homozygotes in the processing of emotional stimuli may explain the interaction between *5-HTTLPR* and stress exposure on ADHD, two of the networks identified by Smith et al. are of particular interest for studying this GxE: the executive control network and the default mode network. The executive control network covers several medial-frontal areas and the basal ganglia, regions richly innervated by serotonergic neurons (Puig and Gulledge 2011) and often linked to ADHD in both task-based and resting-state fMRI (rs-fMRI) analyses (Oldehinkel et al. 2013; Castellanos et al. 2008; Bush et al. 1999). The two brain regions we previously reported to mediate the association of *5-HTTLPR* and stress with ADHD, the frontal pole and anterior cingulate cortex (van der Meer et al. 2015), are also part of this network. It is most strongly activated during cognitive tasks, and is associated with the behavioral domains ‘action-inhibition’, ‘emotion’, and ‘perception-somesthesis-pain’ (Smith et al. 2009). The default mode network consists of inferior lateral and medial parietal regions, as well as the ventromedial frontal cortex. Its activity has been shown to be dependent on serotonin availability (Kunisato et al. 2011). It is the most extensively studied network due to its apparent deactivation during cognitive tasks, sparking discussion on the existence of a default mode of brain functioning (Raichle et al. 2001). This network is associated with self-referential cognitive processes, e.g., using past experiences to plan future actions (Buckner et al. 2008). Heightened default mode network activity and connectivity is associated with negative rumination (Whitfield-Gabrieli and Ford 2012). Lowered suppression of this network during external attention-demanding tasks has been suggested to contribute to problems with cognitive performance of individuals with ADHD, through interfering, task-irrelevant, thoughts leading to lapses of attention (Sonuga-Barke and Castellanos 2007).

In the present study, we aimed to determine whether *5-HTTLPR* genotype moderates the association of stress exposure with functional connectivity in the executive control and default mode networks, and whether connectivity in either of these networks mediate the effect of this GxE on ADHD severity. We therefore performed two mediation analyses, with the GxE as predictor, functional connectivity maps of either the executive control or the default brain network as mediator, and ADHD symptom count as outcome. The analyses were carried out in a sample of adolescents and young adults (mean age 17.2 years) consisting of individuals with and without ADHD, thus enabling analysis within a wide range of ADHD severity, in accordance with the continuous distribution of ADHD within the population (Levy et al. 1997). In this cohort we have previously shown that the interaction between *5-HTTLPR* genotype and stress exposure predicts ADHD severity (van der Meer et al. 2014).

## Materials and methods

Participants were selected from the NeuroIMAGE study, a follow-up of the Dutch part of the International Multicenter ADHD Genetics (IMAGE) study (von Rhein et al. 2014). NeuroIMAGE included 365 families with at least one child with ADHD and at least one biological sibling (regardless of ADHD diagnosis) and 148 control families with at least one child, without any formal or suspected ADHD diagnosis in any of the first-degree family members. ADHD families were recruited through ADHD outpatient clinics in the regions Amsterdam, Groningen, and Nijmegen (the Netherlands). Control families were recruited through primary and high schools in the same geographical regions. To be included in NeuroIMAGE, participants had to be of European Caucasian descent, between ages 5 and 30, have an IQ ≥ 70, and no diagnosis of autism, epilepsy, general learning difficulties, brain disorders, or known genetic disorders. More information on the NeuroIMAGE study and its participants is available elsewhere (von Rhein et al. 2014).

### Measurements

All measurements were part of a comprehensive assessment protocol. Testing was carried out either at the VU University Amsterdam and VU University Medical Centre or at the Radboud University Nijmegen Medical Centre and Donders Institute for Brain, Cognition, and Behavior in Nijmegen. Participants were asked to withhold use of psychoactive drugs for 48 h before measurement. During the testing day, participants were motivated with short breaks and received €50 and a copy of their MRI scan at the end of the day. The study was approved by the regional ethics committee (CMO Regio Arnhem – Nijmegen; 2008/163; ABR: NL23894.091.08) and the medical ethical committee of the VU University Medical Center. All participants signed informed consent (for participants between 12 and 18 years of age, both the parents and participant signed, for participants under 12 only the parents signed).

After selection of those who met the inclusion criteria and had complete phenotypic and good quality rs-fMRI data available, our sample consisted of 425 participants from 255 families. This sample contained 161 participants with a full diagnosis of ADHD, 53 participants with subthreshold ADHD (i.e., ADHD symptoms without meeting the criteria for a full ADHD diagnosis, see the Online resources for a definition), and 207 participants with no ADHD diagnosis. Diagnoses were made in accordance with Diagnostic and Statistical Manual (DSM) IV-TR criteria on the basis of a combination of a semi-structured diagnostic interview, the Kiddie Schedule for Affective Disorders and Schizophrenia - Present and Lifetime version (Kaufman et al. 1997), and the Conners Rating Scales. In this sample, 53 participants had an oppositional defiant disorder or conduct disorder, 12 an internalizing disorder, and 66 reading disorder. An extensive description of the diagnostic algorithm for ADHD and comorbid disorders is provided in the Online resources.

As a measure of ADHD severity, we constructed an ADHD symptom count based on the Conners ADHD Rating Scales questionnaires (Conners et al. 1998). These questionnaires were filled in by the parents and either a teacher (for participants <18 years) or the participants themselves (for those ≥18 years old). The Conners Rating Scales provide operational definitions of each of the 18 ADHD symptoms defined by the DSM-IV-TR. In this sample, the symptom count ranged from 0 to 18 with an average of 5.1 (standard deviation (SD) 5.0). For more details on how this measure was constructed, see the Online resources.

Two questionnaires were used to assess the amount of exposure to psychosocial stress, as described in previous work (van der Meer et al. 2014). Parents filled in the Long-Term Difficulties (LTD) questionnaire (Bosch et al. 2012; Oldehinkel et al. 2008), which contained thirteen items measuring whether their children have been exposed to chronic stressors such as a handicap, being bullied, having financial difficulties, or other persisting problems at home or school. They were asked to only report chronic, ongoing difficulties. In addition, participants themselves filled in a Stressful Live Events (SLE) questionnaire (Bosch et al. 2012; Oldehinkel et al. 2008), which contained eleven items on exposure to specific major stressful events in the past 5 years, such as death or serious illness of a loved one, physical or sexual abuse, or failure at something important to them. For the composite stress measure, the scores on these questionnaires were transformed to Z-values and averaged according to common practice for aggregating similar measures, as previously described elsewhere (van der Meer et al. 2014). See the Online resources for further information on both questionnaires and an overview of the items.

Genotyping was performed as described in Brookes et al. (2006) and in the Online resources. Briefly, DNA was extracted from blood samples at Rutgers University Cell and DNA Repository, New Jersey, USA. Standard polymerase chain reaction protocols were used for the determination of *5-HTTLPR* genotype. This study investigated a dominant genetic model of the *5-HTTLPR* S-allele, wherein S-allele carriers were coded as ‘1’ and L-allele homozygotes were coded as ‘0’. This is in accordance with the majority of studies investigating this GxE (Caspi et al. 2010) and is based on the functional effects of the S- and L-alleles (Lesch et al. 1996). In addition, L-alleles with the rs25531 C-G single nucleotide polymorphism were recoded as a functional S-allele, in accordance with prior studies (Hu et al. 2006). This led to 20 L-allele homozygotes being recoded to S-allele carriers. Genotype frequencies did not deviate from Hardy-Weinberg Equilibrium (χ^2^ = 0.66, *p* = .42).

As a measure of socio-economic status, the highest successfully completed education level of the parents was recoded into a measure reflecting years of education. This scale contained nine levels, ranging from 0 (no formal education) to 17 (university) years of education (Buis 2010). The average of both parents was used, which, in this sample, ranged from 5 to 17 with an average of 12.1 (SD 2.5).

### Resting-state fMRI data processing

All subjects were scanned with either a Siemens MAGNETOM Sonata 1.5 Tesla (at VU UMC in Amsterdam) or a Siemens MAGNETOM Avanto 1.5 Tesla (at Donders Centre for Cognitive Neuroimaging in Nijmegen) MRI scanner (Siemens, Erlangen, Germany), using identical protocols. Functional images during rest were obtained using a gradient echo echo-planar imaging (GE-EPI) sequence (TR = 1960 ms, TE = 40 ms, FOV = 224 mm, 37 axial slices, flip angle =80, matrix size =64 × 64, in-plane resolution =3.5 mm, slice thickness/gap =3.0 mm/0.5 mm, 266 volumes). Participants were instructed to relax with their eyes open during the rs-fMRI scan, which lasted 8 minutes.

Preprocessing consisted of removal of the first five volumes, primary head movement correction by realignment to the middle volume, global 4D mean intensity normalization and spatial smoothing with a 6 mm Gaussian kernel. We corrected for secondary effects of head motion by applying ICA-AROMA, a robust ICA-based strategy for automatic detection and removal of motion-related artifacts (Pruim et al. 2015). We additionally removed signal from white matter and cerebrospinal fluid using nuisance regression, and applied temporal high-pass filtering (>0.01 Hz). We co-registered the functional data to the participant’s structural image using affine boundary-based registration as implemented in FSL FLIRT (Jenkinson et al. 2002) and subsequently transformed them to MNI152 standard space with 4 mm isotropic resolution using non-linear registration through FSL FNIRT.

Starting out with 486 participants who had behavioral and rs-fMRI data available, we first excluded the top 5 % of participants with highest motion score (*n* = 24), as calculated by the root mean squared of the frame-wise displacement time series. Subsequently, 19 individuals were removed based on scan quality (e.g. artifacts, less than 240 volumes available, insufficient coverage of the entire brain), and 18 due to incidental findings after visual inspection (e.g. enlarged ventricles or unexpected hypo-intensities), for our final sample size of *n* = 425.

For each participant, we derived spatial maps of the executive control and default mode networks using dual regression (Beckmann et al. 2009). This consisted of a multivariate spatial regression of a set of initial templates against the preprocessed rs-fMRI data, yielding participant-specific time series. The resulting time series were then entered in a multivariate temporal regression against the same preprocessed rs-fMRI data resulting in participant-level spatial representations of the initial templates (Beckmann et al. 2009; Filippini et al. 2009).

As initial template set, we used the twenty-dimensional resting-state components described by Smith et al. (2009), which have been made publicly available. We chose these because they have been shown to closely correspond to brain networks identified in thousands of individuals across a wide range of tasks (Smith et al. 2009). Use of these templates allows for better comparison between studies and comes with a large amount of information on the networks’ behavioral correlates from previous studies (Laird et al. 2011; Smith et al. 2009).

### Mediation analyses

For the mediation analyses, we employed Mediation Effect Parametric Mapping (Wager et al. 2008). This analysis technique is based on a standard three-variable mediation model, investigating the association of the predictor X with the mediator M, and the association of mediator M with the dependent variable Y. The mediation effect, i.e., the effect of X on Y mediated by M, is the product of the resulting two regression coefficients, the significance of which is determined through bootstrapping (Hayes 2013). We ran two mediation models, one for either network. Both consisted of *5-HTTLPR* genotype, amount of stress exposure, and their interaction as predictors, the map resulting from the output of the dual regression on the executive control or default mode network template as mediator, and ADHD symptom count as dependent variable. We included sex, age, scanner location, and socio-economic status as covariates. All predictors and covariates were mean-centered. The mediation analyses were performed in MATLAB with the Multilevel Mediation and Moderation Toolbox (Wager et al. 2008), which performed bootstrapping (5000 samples) on each voxel. Family-wise error correction was applied through the use of FSL’s EasyThresh, which carries out cluster-based thresholding. A Z-value of 2.3 was used to define contiguous clusters and subsequently, each cluster’s significance level was estimated on the basis of Gaussian Random Field theory. Those clusters surviving a significance threshold of *p* = .01 are reported. We used the Harvard-Oxford atlas for localization. All reported coordinates are in MNI-space and in millimeter.

For plotting of the results and calculation of summary statistics reported in Table 2, we used R, v3.1.1. (R CoreTeam 2012). For every participant we extracted the mean network connectivity from the clusters identified in the main analyses. We reran the analyses with linear mixed effects models, estimating a random intercept for family. In addition to *5-HTTLPR*, stress exposure, and their interaction, all models had sex, age, scanner location, and socio-economic status included as covariates. We calculated Cohen’s f^2^ as a measure of effect size for the significant predictors (Selya et al. 2012), see Table 2.

### Sensitivity analyses

We checked whether the direction of effects was the same across diagnostic status, testing locations, and age groups, to ensure findings were not driven by any one group. More information on the methods for these analyses can be found in the Online resources.

## Results

No significant differences in stress exposure, sex, age, testing location, socio-economic status or head motion during scanning were found between S-allele carriers and L-allele homozygotes, as summarized in Table 1.

**Table 1:** Demographic information on the participants, split by *5-HTTLPR* genotype

Variable	S-allele carriers	SD	L-allele homozygotes	SD	Test-statistic	DF	*P*-value
Participants	284		141				
Covariates							
Amsterdam location	49.3 %		57.4 %		Χ^2^ = 2.51	1	.11
Male sex	53.5 %		62.4 %		Χ^2^ = 2.68	1	.10
Age in years	17.19	3.49	17.44	3.41	F = 0.47	423	.49
Parents’ years of education	11.92	2.50	12.29	2.50	F = 1.99	423	.16
Stress score	1.55	1.18	1.75	1.17	F = 2.78	423	.10
Number of stressful live events	2.00	1.52	2.21	1.56	F = 1.76	423	.19
Number of long-term difficulties	1.03	1.41	1.29	1.50	F = 3.00	410	.08
Head motion during scanning	0.67	2.18	0.79	2.75	F = 0.26	423	.61

Differences between genotypes in the categorical variables ‘location’ and ‘sex’ were analyzed with a Chi-square test; for the other, continuous variables we performed an analysis of variance. *SD* standard deviation, *DF* degrees of freedom. Head motion was measured as the root mean squared of the frame-wise displacement time series

*5-HTTLPR* genotype significantly moderated the effect of stress exposure on ADHD symptom count (B = 1.65, SE = 0.61, *p* = .007), as previously reported in a sample from which the current sample is a subset (van der Meer et al. 2014). The conditional effects of genotype or stress exposure on ADHD symptom count were not significant. Within-group analysis confirmed that stress exposure was highly significantly related to ADHD symptom count in S-allele carriers (B = 2.07, SE = 0.37, *p* < .0001), but not in L-allele homozygotes (B = 0.58, SE = 0.53, *p* = .28).

### Executive control network connectivity

The executive control network covers large portions of the medial frontal lobe, frontal pole, and basal ganglia. The corresponding brain map is displayed in the Online resources.

**Fig. 1:**
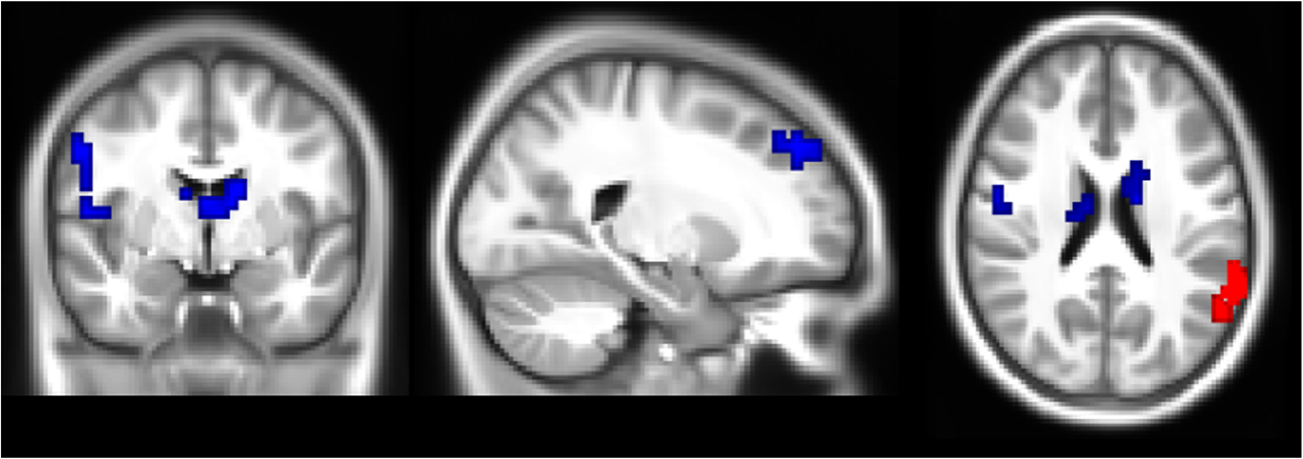
Location of clusters where the *5-HTTLPR* by stress interaction had a significant negative effect on connectivity in the executive control network (*blue*), and a positive effect on connectivity in the default mode network (*red*). These maps are overlaid on the sample’s average anatomical image, at MNI-coordinates 22, −2, 22

**Fig. 2:**
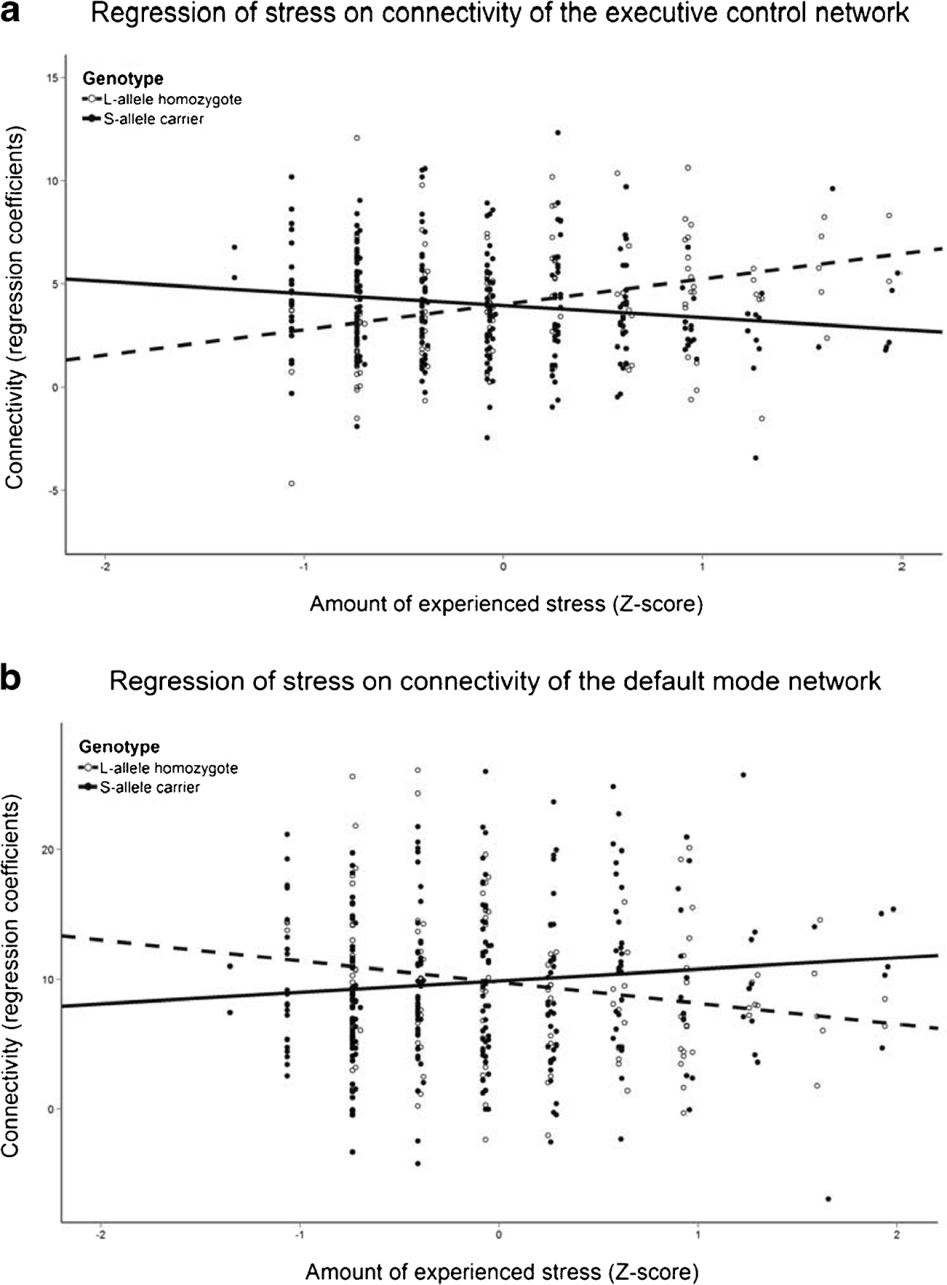
Interaction effect between *5-HTTLPR* genotype and stress exposure on connectivity, with S-allele carriers represented by the solid line and points and L-allele homozygotes by the dashed line and open points. **a** displays the mean of the regression coefficients from the four clusters found in the executive control network, and **b** shows the interaction effect found for the cluster in the default mode network

The association between stress and connectivity of regions in the executive control network was moderated by *5-HTTLPR* genotype bilaterally in the precentral gyrus extending into the postcentral gyrus, right frontal pole, left thalamus and caudate nucleus (see Fig. 1). S-allele carriers had a more negative correlation between stress and connectivity than L-allele homozygotes in all four clusters (see Fig. 2A and Table 2).

**Table 2:** Information on the clusters where resting-state network connectivity was found to be significantly associated with the gene-environment interaction

RSN	Location	X	Y	Z	Cluster size	Coefficient	Cohens f^2^
Executive	Post-, precentral gyrus	-34	-22	36	21	-1.57	.023
	Frontal pole	22	46	40	26	-2.61	.035
	Post-, precentral gyrus	54	-2	36	47	-2.33	.036
	Caudate nucleus, thalamus	-10	-2	16	68	-2.14	.050
DMN	Supramarginal gyrus	-62	-50	24	22	2.62	.033

X, Y, Z coordinates are in MNI-space in mm, and represent the peak of the cluster. The anatomical labels are according to the Harvard-Oxford atlas. *RSN* Resting-state network, *DMN* Default-mode network, *MNI* Montreal Neurological Institute. Cluster size indicates number of voxels in that cluster, at 4 mm isotropic resolution

### Default mode network connectivity

The default mode network covers large portions of the posterior cingulate and precuneus, angular gyrus, and frontal medial cortex. The corresponding brain map is displayed in the Online resources.

In the default mode network, the effect of stress was moderated by *5-HTTLPR* genotype in the left posterior supramarginal gyrus extending into the angular gyrus (see Fig. 1 and Table 2). In this region, S-allele carriers had a more positive correlation between stress and connectivity than L-allele homozygotes (see Fig. 2B).

We did not find any mediation effects, i.e. connectivity patterns in either network did not significantly explain the association between the GxE and ADHD severity.

Given our focus on the GxE, details on the conditional effects of stress and genotype on connectivity, and the association between connectivity and ADHD severity, are presented in the Online resources.

### Sensitivity analyses

Results from the sensitivity analyses can also be found in the Online resources. Briefly, the direction of effects was the same across diagnostic status, testing locations, and age groups.

## Discussion

We investigated whether the interaction between *5-HTTLPR* genotype and stress exposure is associated with differences in connectivity of two brain networks involved in the processing of emotional stimuli, the executive control network and default mode network. We combined this with mediation analysis to determine whether connectivity of these networks could explain a previously reported significant association between this GxE and ADHD severity (van der Meer et al. 2014).

In the executive control network, S-allele carriers had a more negative association between the amount of stress exposure and functional connectivity than L-allele homozygotes, whereas they had a more positive relation between stress exposure and connectivity of the posterior hub of the default mode network. While speculative, the reported effects may contribute to the often-reported association of this GxE with neuroticism, rumination, and worrying (Lesch et al. 1996). Higher default mode network connectivity during rest has been tied directly to greater trait rumination (Berman et al. 2011), and lowered cognitive control is also associated with rumination and worrying (Beckwe et al. 2014). The opposing pattern in the two networks is particularly interesting in light of findings suggesting that the anti-correlation between task-positive and task-negative networks is of higher relevance to behavior than network activity per se (Fox et al. 2005). Specifically, dominance of regions involved in internally-oriented attention over regions involved in externally-oriented attention has been proposed to underlie rumination, impaired attentional control, and cognitive reactivity (Marchetti et al. 2012), and an imbalance between default mode and regulatory networks has been repeatedly found in psychiatric populations characterized by emotional problems (Belleau et al. 2014). Our results may therefore reflect the tendency for S-allele carriers to develop a dysfunctional, internally-focused, cognitive style when exposed to stress (Wells et al. 2010).

We found lowered functional connectivity of the precentral gyrus extending into the postcentral gyrus, frontal pole, and caudate nucleus with the executive control network, all regions previously associated with this GxE (Canli et al. 2005, 2006; van der Meer et al. 2015) and all important for cognitive control when faced with emotional stimuli. The precentral gyrus is active during cognitive reappraisal of distressing stimuli (Belden et al. 2014). The postcentral gyrus has shown higher activity specifically during the presentation of negative words in an emotional Stroop task, in a study that showed that tryptophan depletion (i.e., lowered serotonin signaling) improved task performance (Evers et al. 2006). The frontal pole is central to inhibiting automatic emotional responses (Volman et al. 2011). And lastly, connectivity of the caudate nucleus with the prefrontal cortex has been found to explain successful emotion regulation through reappraisal (Wager et al. 2008). We further found a significantly more positive relation between stress exposure and connectivity of the supramarginal gyrus within the default mode network for S-allele carriers compared to L-allele homozygotes, in line with previous reports of effects of this GxE on the superior parietal lobe (Canli and Lesch 2007). Heightened connectivity of this region may be tied to its role in self-referential processing (Silani et al. 2013), given the association of *5-HTTLPR* and stress exposure with rumination and worrying (Clasen et al. 2011; Schinka et al. 2004), traits common in individuals with ADHD (Nigg et al. 2002; Shaw et al. 2014). The lateral parietal lobe is also linked to post-traumatic stress disorder (Morey et al. 2011; Liberzon and Sripada 2007) and known to facilitate episodic memory retrieval (Davidson et al. 2008), supporting the notion that this finding relates to rumination.

The reported connectivity patterns did not significantly explain why S-allele carriers have a stronger association between stress exposure and ADHD severity than L-allele homozygotes. Whereas we previously reported that gray matter volume in the frontal pole mediated the effects of this GxE on ADHD (van der Meer et al. 2015), functional connectivity of this region within the executive control network did not. Lack of significant mediation findings in this study indicates that the effect of this GxE on connectivity patterns within either network is insufficient to explain its relation with ADHD severity. It may be the case though, that a combination of connectivity patterns within several networks is necessary to significantly contribute to behavior as complex as the ADHD phenotype. For instance, interference of task-irrelevant thoughts through decreased suppression of default mode network activity has been suggested to contribute to lower cognitive performance of individuals with ADHD (Sonuga-Barke and Castellanos 2007), which may result both from lowered connectivity of the executive control network and stronger connectivity of the default mode network. This combined effect may be investigated in future studies analyzing cross-network connectivity. It may also be captured during task performance taxing both cognitive control and emotion processing. The emotional Stroop task seems particularly suitable for this, as the brain regions found in the current study have all been reported to be active during this task (Veroude et al. 2013; Evers et al. 2006; Sadeh et al. 2011; Malhi et al. 2005). In addition, *5-HTTLPR* genotype has been shown to influence recruitment of cognitive control brain regions during incongruent trials (Stollstorff et al. 2013), and individuals with ADHD have shown higher interference by negatively-valenced words compared to healthy controls (Posner et al. 2011).

Strengths of this study include a large sample size, use of multiple informants to determine ADHD phenotype, and the hypothesis-driven study of robust and reliable brain networks using multivariate statistics. A limitation is the observational, cross-sectional design of our study, preventing strong inferences about causality. For instance, it could be the case that the reported neural differences produce maladaptive behavior, which in turn may lead to the experience of more stressful live events. While animal studies have provided causal evidence for the brain of S-allele carriers being more strongly affected by exposure to stress (Spinelli et al. 2007), longitudinal studies or studies making use of ‘natural experiments’ (Rutter 2007), are needed to confirm this causality in humans.

In conclusion, the interaction between *5-HTTLPR* and stress exposure is associated with decreased connectivity in the executive control network and increased connectivity within the default mode network. Although in need of replication, the reported effects may contribute to the association of this GxE with a range of pathological behaviors due to lowered cognitive control and enhanced emotional reactivity. Ultimately, this type of research may contribute to the prevention and treatment of stress-related pathological behavior, by identifying those most at risk and by providing knowledge on the neural mechanisms involved.

## Electronic supplementary material

Online Resources 1 (DOC 637 kb)
