# Supplementary material for: The interaction between 5-HTTLPR and stress exposure influences connectivity of the executive control and default mode brain networks
Source: Brain Imaging Behav. 2016 Oct 13;11(5):1486–96. doi: 10.1007/s11682-016-9633-3 (PMC5653701; doi:10.1007/s11682-016-9633-3)
Supplement: Supplementary file 1 — (DOC 637 kb) [file 11682_2016_9633_MOESM1_ESM.doc]

**Supplementary Information**

**ADHD diagnostic algorithm**

To determine psychiatric diagnoses, all participants (children and parents alike) were assessed with a combination of ADHD rating scales and a semi-structured diagnostic interview. In order to determine ADHD diagnoses, a diagnostic algorithm was applied based on the behavioral questionnaires (typically filled in by parents as well as a second observer) and the diagnostic interview, using DSM-IV criteria (American Psychiatric Association, 2000). Inconsistent cases were reviewed by a team of trained experts, in order to derive a consensus diagnosis.

**Measures**

*Children* were assessed with a parent rating scale (CPRS-R:L; 1998a), and either a teacher rating scale (CTRS-R:L; 1998b), applied for children < 18 years, or a self-report (CAARS-S:S; 1999), applied for children ≥ 18 years. A semi-structured diagnostic interview (KSADS-PL; Kaufman et al., 1997) was administered to both the children (if ≥ 12 years old) and their parents separately. Initially, all participants were only administered the screening interview. Participants with elevated scores on any of the screen items were administered the full ADHD section.

*Parents* were assessed similarly with an observer ADHD rating scale (CAARS-O:SV; 1999), typically filled in by their partner. The KSADS-PL was administered to all parents, who were, if possible, interviewed together with their partner.

Of the Conners' ADHD questionnaires the following scales were used:

- DSM Inattentive behavior
- DSM Hyperactive/Impulsive behavior
- DSM Total

For all participants using medication, ratings were done of the participant's functioning off medication.

**The diagnostic algorithm**

The diagnostic algorithm applied to all participants was based on a combination of symptom counts on the ADHD rating scales and the KSADS-PL, both providing operational definitions of each of the 18 behavioral symptoms of ADHD defined by the DSM-IV. Combined counts for each symptom were determined based on the KSADS-PL scores combined with scores on either the teacher rating scale (for children <18 years), the self-report (for children ≥18), or the observer rating (for parents).

Based on the algorithm, participants were given either an 'affected' (ADHD diagnosis) status or 'unaffected' status.

The following criteria were used to classify ADHD ('affected' status):

- Combined symptom count of ≥ 6 symptoms of inattentive or hyperactive/impulsive behavior
- T-score ≥ 63 on at least one of the ADHD subscales on at least one of the available Conners' ADHD rating scales
- Age of onset before 12
- Symptoms cause clinical impairment
- Symptoms are not better accounted for by another disorder

For children ≥18 years and parents, criteria were slightly adapted, such that a combined symptom count of 5 symptoms and age of onset before 15 years were sufficient for an 'affected' status.

Participants were labelled 'unaffected' if they received a T<63 on each of the scales of the Conners' rating scales, and if they had ≤ 3 symptoms (or ≤ 2 symptoms for children of ≥18 years and parents), derived from the combined symptom counts.

For analysis purposes, participants who did not meet criteria for either affected or unaffected status, were labelled 'subthreshold ADHD'.

**Comorbid disorders**

Participants were diagnosed with ODD if they exhibited four or more of the DSM-IV symptoms derived from the K-SADS. Likewise, conduct disorder (CD) was determined if a participant exhibited three symptoms or more DSM-IV symptoms derived from K-SADS interviews.

For internalizing disorders, we used the anxiety and depression module of the K-SADS, which was administered if the participants had elevated scores on the screening section. Diagnoses were made based on the instructions given therein, in accordance with DSM-IV-TR criteria.

Reading disorder was not diagnosed directly within the NeuroIMAGE project, but pre-existing diagnosis of reading disorder by a recognized medical institution were incorporated in the study design.

**References**

American Psychiatric Association. (2000). *Diagnostic and statistical manual of mental disorders: DSM-IV-TR*: American Psychiatric Publishing, Inc.

Conners, C. K., Erhardt, D., & Sparrow, E. P. (1999). *Conner's Adult ADHD Rating Scales: CAARS*: Multi-Health Systems, North Tonawanda, NY.

Conners, C. K., Sitarenios, G., Parker, J. D. A., & Epstein, J. N. (1998a). The revised Conners' Parent Rating Scale (CPRS-R): factor structure, reliability, and criterion validity. *Journal of abnormal child psychology, 26*(4), 257-268.

Conners, C. K., Sitarenios, G., Parker, J. D. A., & Epstein, J. N. (1998b). Revision and restandardization of the Conners Teacher Rating Scale (CTRS-R): factor structure, reliability, and criterion validity. *Journal of abnormal child psychology, 26*(4), 279-291.

Kaufman, J., Birmaher, B., Brent, D., Rao, U., Flynn, C., Moreci, P., et al. (1997). Schedule for Affective Disorders and Schizophrenia for School-Age Children-Present and Lifetime Version (K-SADS-PL): initial reliability and validity data. *J Am Acad Child Adolesc Psychiatry, 36*(7), 980-988.

**Assessment of stress exposure**

*The long-term difficulties questionnaire*

This questionnaire was filled in by the participants’ parents with the instruction to indicate which situations are currently applicable to their child.

1. Your child has a chronic illness or handicap.
2. Someone else in the immediate family has a chronic illness or handicap.
3. Your child has a very high work pressure at school.
4. There are issues with your house (for instance too small, noisy, or busy).
5. There are issues with your neighborhood (for instance vandalism, unsafe).
6. Someone in your immediate family lost their job or became unfit for work for longer than three months.
7. Your immediate family has financial difficulties.
8. Your child has fewer friends than he/she would like.
9. Your child is being bullied at school or in the neighborhood.
10. Your child can’t get along with someone in your immediate family.
11. Your child can’t get along with someone else.
12. Other immediate family members can’t get along with each other.
13. Your partner and you are separated.

*The stressful live events questionnaire*

This questionnaire was filled in by the participants themselves with the instructions to indicate whether they experienced any of the following events in the past five years.

1. A romantic relationship ended against your will.
2. An important friendship ended.
3. You failed on something important to you.
4. A change occurred affecting your immediate family, making it a lot less pleasant there.
5. A change occurred at school or work, making it a lot less pleasant there.
6. A group of friends whom you spent a lot of time with wanted nothing to do with you anymore.
7. You left a church or religious community because you didn’t feel at home there anymore.
8. A loved one died.
9. You were so seriously ill that there were concerns of permanent consequences or death.
10. You were physically abused.
11. You were raped or sexually assaulted.

*Composite stress measure*

If participants filled in less than half the items on both questionnaires, they were excluded from further analysis; (2.1% missing data for the SLE score; 3.3% for the LTD score). If more than half the items were filled in, missing items were imputed with ‘no’, i.e. we assumed the major life event had not occurred if not reported; 0.6% of the items were imputed for the SLE and 3.6% of the items for the LTD questionnaire. After this, the scores on the questionnaires were transformed to Z-values and averaged according to common practice for aggregating similar measures.

**DNA extraction and genotyping**

For the IMAGE sample DNA was extracted from blood samples or immortalized cell lines at Rutgers University Cell and DNA Repository, New Jersey, USA as well the Human Genetics department of the Radboud University Medical Centre in Nijmegen. Additional NeuroIMAGE samples were collected in the form of a saliva sample. DNA was isolated from saliva using Oragene containers (DNA Genotek, Ottawa, Ontario, Canada) according to the protocol supplied by the manufacturer at the Radboud’s department of Human Genetics.

Genome-wide genotyping for IMAGE was performed as part of the GAIN study using the Perlegen 600K genotyping platform, which comprises approximately 600,000 tagging single nucleotide polymorphisms (SNPs; . For NeuroIMAGE samples not genotyped during IMAGE genotyping was performed on the Human CytoSNP-12 version 2 genotyping BeadChip (Illumina Inc., San Diego, California, USA) at the Lifelines facility (Groningen, The Netherlands). Quality control steps were performed for the genotype data. SNPs were excluded if the call rate per SNP was less than 95%, the minor allele frequency was less than 1%, or the SNPs failed the Hardy-Weinberg equilibrium test at a threshold of p≤10–6 (genome-wide). Participants were excluded if the call rate per individual was lower than 95%.

Genotyping of the *HTTLPR* variable number tandem repeat (VNTR) polymorphism had been performed previously by the IMAGE consortium (50). Standard PCR protocols were used for all VNTR markers and amplified products were visualized on 2% agarose under UV light. For the additional NeuroIMAGE samples, the VNTR was genotyped using standard PCR protocols. After the PCR, fragment length analysis was performed on the ABI Prism 3730 Genetic Analyser (Applied Biosystems, Nieuwekerk a/d Ijssel, The Netherlands) and results were analyzed with GeneMapper® Software, version 4.0 (Applied Biosystems).

**Sensitivity Analyses**

*Direction of effects within subsamples, based on diagnosis, location, and age*

As a check, we reran the analyses on the significant findings from the main analysis within diagnostic groups (control subjects and those with subthreshold ADHD combined, separately from those with an ADHD diagnosis), as well as within both locations (Amsterdam and Nijmegen), and within children (<18 years) and adults (≥ 18 years) separately. The purpose of this was to ensure the results were not strongly influenced by any particular subsample, by checking whether the direction of effects is the same across groups. The results of these analyses are summarized in Table S2.

*Table S2. Direction of effects within the subsamples for significant findings from the main analysis. The regression coefficients refer to that of the gene-environment interaction term for each subset.*

| *Region* | *Full* | *Nijmegen* | *Amsterdam* | *Controls* | *ADHD* | *<18 years* | *≥18 years* |
| --- | --- | --- | --- | --- | --- | --- | --- |
| Postcentral gyrus | -1.57 | -1.85 | -1.61 | -2.09 | -0.79 | -1.44 | -0.96 |
| Frontal pole | -2.61 | -3.52 | -1.90 | -2.41 | -3.14 | -2.04 | -2.57 |
| Postcentral gyrus | -2.33 | -1.48 | -3.10 | -2.97 | -1.62 | -2.27 | -0.57 |
| Thalamus | -2.14 | -3.41 | -1.09 | -2.08 | -2.37 | -1.23 | -2.72 |
| Supramarginal gyrus | 2.62 | 2.07 | 3.45 | 2.34 | 3.36 | 2.19 | 2.43 |
| Symptoms | 1.65 | 1.23 | 2.15 | 0.69 | 1.78 | 1.84 | 1.31 |

**Full results from the whole-brain mediation analyses**

*Table S3. Summary of the clusters where genotype, stress exposure, and the gene-environment interaction are significantly correlated with executive control network connectivity at p=.01, as determined by Random Field Theory.*

Predictor: Stress

| *Path* | *Direction* | *Location* | *X* | *Y* | *Z* | *Cluster size* | *Z_Max_* |
| --- | --- | --- | --- | --- | --- | --- | --- |
| A | Positive | Central opercular cortex | 50 | -14 | 12 | 32 | 3.36 |
|  | Positive | Thalamus | 2 | -18 | 8 | 19 | 3.49 |
|  | Positive | Post,- precentral gyrus | -42 | -26 | 40 | 18 | 3.58 |
|  | Negative | Postcentral gyrus, Supramarginal gyrus | 38 | -38 | 44 | 16 | 3.3 |
| AB | N/A | N/A |  |  |  |  |  |

Predictor: Genotype

| *Path* | *Direction* | *Location* | *X* | *Y* | *Z* | *Cluster size* | *Z_Max_* |
| --- | --- | --- | --- | --- | --- | --- | --- |
| A | L>S | Precentral gyrus | 6 | -30 | 64 | 40 | 3.43 |
|  | L>S | Supplementary motor cortex | 2 | -10 | 52 | 32 | 3.47 |
|  | L>S | Frontal pole | -34 | 54 | 24 | 25 | 3.21 |
|  | L>S | Superior frontal gyrus | -18 | 6 | 32 | 20 | 3.48 |
| AB | N/A | N/A |  |  |  |  |  |

Predictor: GxE

| *Path* | *Direction* | *Location* | *X* | *Y* | *Z* | *Cluster size* | *Z_Max_* |
| --- | --- | --- | --- | --- | --- | --- | --- |
| A | Negative | Post-, precentral gyrus | -34 | -22 | 36 | 21 | 3.41 |
|  | Negative | Frontal pole | 22 | 46 | 40 | 26 | 3.41 |
|  | Negative | Post-, precentral gyrus | 54 | -2 | 36 | 47 | 3.45 |
|  | Negative | Thalamus, caudate nucleus | -10 | -2 | 16 | 68 | 3.42 |
| B* | Positive | Paracingulate, Anterior cingulate gyrus | 14 | 54 | 18 | 169 | 3.60 |
|  | Positive | Middle frontal gyrus | 54 | 26 | 32 | 21 | 3.50 |
| AB | N/A | N/A |  |  |  |  |  |

*Table S4. Summary of the clusters where genotype, stress exposure, and the gene-environment interaction are significantly correlated with default mode network connectivity at p=.05, as determined by Random Field Theory.*

Predictor: Stress

| *Path* | *Direction* | *Location* | *X* | *Y* | *Z* | *Cluster size* | *Z_Max_* |
| --- | --- | --- | --- | --- | --- | --- | --- |
| A | Negative | Subcallosal cortex | -10 | 22 | -20 | 25 | 3.43 |
|  | Negative | Angular gyrus | 58 | -58 | 28 | 15 | 3.76 |
| AB | N/A | N/A |  |  |  |  |  |

Predictor: Genotype

| *Path* | *Direction* | *Location* | *X* | *Y* | *Z* | *Cluster size* | *Z_Max_* |
| --- | --- | --- | --- | --- | --- | --- | --- |
| A | L > S | Occipital fusiform gyrus | 34 | -82 | -8 | 26 | 3.2 |
|  | L > S | Superior frontal gyrus | -22 | 30 | 44 | 25 | 3.41 |
|  | L > S | Frontal medial cortex, Subcallosal cortex | 2 | 34 | -12 | 25 | 3.36 |
|  | S > L | Cerebellum Left Crus II | -38 | -58 | -48 | 24 | 3.54 |
| AB | N/A | N/A |  |  |  |  |  |

Predictor: GxE

| *Path* | *Direction* | *Location* | *X* | *Y* | *Z* | *Cluster size* | *Z_Max_* |
| --- | --- | --- | --- | --- | --- | --- | --- |
| A | Positive | Supramarginal gyrus, Angular gyrus | -62 | -50 | 24 | 22 | 3.64 |
| B* | Positive | Supramarginal gyrus | -46 | -54 | 16 | 19 | 3.51 |
| AB | N/A | N/A |  |  |  |  |  |

*Note: X, Y, Z coordinates are in MNI-space and represent the peak of the cluster. The anatomical labels are according to the Harvard-Oxford atlas. MNI=Montreal Neurological Institute; Z_max_= Z-score at the peak of the cluster. Path A represents the correlation between the predictors and GMV, *path B represents the correlation between GMV and ADHD symptom count (this path is the same for genotype, stress, and the GxE term, and is therefore only displayed in the GxE part of the table), and path AB represents the mediation analysis.*

**Resting state networks**

Figure S1 shows the executive control (top) and default mode (bottom resting state networks, as identified by Smith *et al.* (2009). These maps can be downloaded at <http://www.fmrib.ox.ac.uk/analysis/brainmap+rsns/>

*Executive control network*


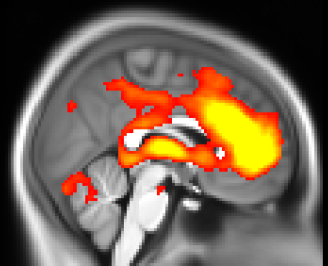

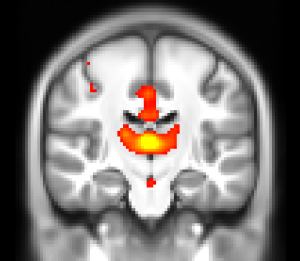

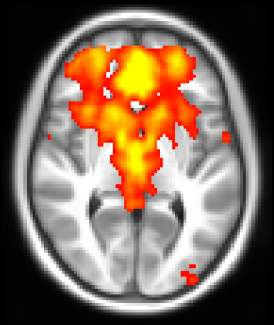


*Default mode network*


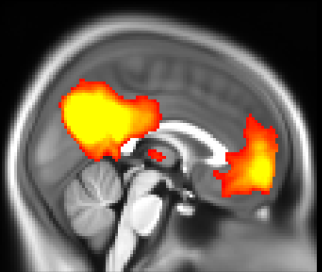

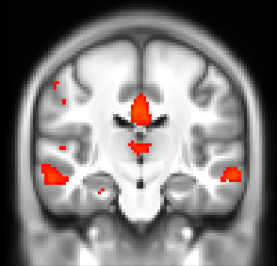

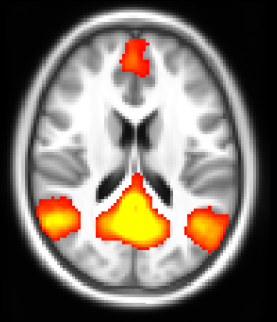


*Figure S1. Resting-state networks as identified through independent component analysis by Smith et al. (2009). The data is overlain on the study sample’s average anatomical image at MNI-coordinates X=2, Y=-18, Z=4.*
